# Melodic contour supersedes short-term statistical learning in expressive accentuation

**DOI:** 10.1371/journal.pone.0312883

**Published:** 2024-11-25

**Authors:** Haiqin Zhang, Emmanuel Chemla, Claire Pelofi, Laurent Bonnasse-Gahot

**Affiliations:** 1 Centre d’Analyse et de Mathématique Sociales, École des Hautes Études en Sciences Sociales, CNRS, Paris, France; 2 Département des Études Cognitives, École Normale Supérieure Paris, Paris Sciences et Lettres University, Paris, France; 3 Laboratoire de Sciences Cognitives et Psycholinguistique, École Normale Supérieure Paris, Paris Sciences et Lettres University, Paris, France; 4 Center for Language, Music and Emotion, New York University and the Max Plank Institute for Empirical Aesthetics, New York, NY, United States of America; University of Missouri Columbia, UNITED STATES OF AMERICA

## Abstract

Sensory systems are permanently bombarded with complex stimuli. Cognitive processing of such complex stimuli may be facilitated by accentuation of important elements. In the case of music listening, alteration of some surface features –such as volume and duration– may facilitate the cognitive processing of otherwise high-level information, such as melody and harmony. Hence, musical accents are often aligned with intrinsically salient elements in the stimuli, such as highly unexpected notes. We developed a novel listening paradigm based on an artificial Markov-chain melodic grammar to probe the hypothesis that listeners prefer structurally salient events to be consistent with salient surface properties such as musical accents. We manipulated two types of structural saliency: one driven by Gestalt principles (a note at the peak of a melodic contour) and one driven by statistical learning (a note with high surprisal, or information content [IC], as defined by the artificial melodic grammar). Results suggest that for all listeners, the aesthetic preferences in terms of surface properties are well predicted by Gestalt principles of melodic shape. In contrast, despite demonstrating good knowledge of novel statistical properties of the melodies, participants did not demonstrate a preference for accentuation of high-IC notes. This work is a first step in elucidating the interplay between intrinsic, Gestalt-like and acquired, statistical properties of melodies in the development of expressive musical properties, with a focus on the appreciation of dynamic accents (i.e. a transient increase in volume). Our results shed light on the implementation of domain-general and domain-specific principles of information processing during music listening.

## Introduction

Music listening is an inherently active process by which listeners tend to continuously form musical expectations, which are informed by their long-term exposure throughout the lifetime (i.e., musical culture) and the short-term context of a musical stimulus [1]. In recent years, various models have been used to explain, to a great extent, the mechanisms by which listeners use musical expectations predict the next notes in a melody [2–4], and how these melodic features influence aesthetic preferences. Some of this research has focused on stimuli generated from symbolic representations (e.g., MIDI files), which allows surface properties to be extracted from the written score (e.g. note length, dynamic markings) [5]. Other works have studied the use of expressive, *performed* accents: accents that are not explicitly specified in the notated score, but are interpretation decisions made by the performer. The performer might choose to emphasise a particular note such by playing it at a louder volume (dynamic accent), or slightly changing the timing or articulation (temporal accent) [5, 6].

Arguably, this expressive accenting is tightly linked to melodic expectations, allowing important elements to be highlighted and thereby segmenting music into digestible fragments for cognitive processing [7]. Previous work has shown that performed accents are aligned with various types of *immanent* accents –i.e. salient events revealed by analysis of the musical structure, that naturally enhance particular notes in a given context– and are sometimes explicitly marked on the music score. For example, temporal and dynamic accents are aligned with rhythmic groupings [7], and statistically surprising chords are played at a higher volume and for a longer duration across a large cross-section of the Western classical music corpus [5].

Immanent accents originate from two distinct types of processes: statistical regularities –which result from an arbitrary and culture-specific sets of rules that are internalised by listeners through long-term passive exposure– and Gestalt principles –which govern structural properties of auditory sequences at large. Statistical regularities are culture-specific and learnt through exposure. Musical systems vary around the world, employing different sets of pitches, harmonies, and transitional probabilities between them [2]. Therefore, listeners exposed to a particular musical culture acquire, through statistical learning, an internal grammar that reflects these structural regularities. In contrast, Gestalt principles constitute a simple and somewhat limited set of rules. For example, the principle of proximity posits that melodies are more coherent when intervals between pitches are small. An immanent accent results from a large leap which makes a note less coherent, and thus more salient [8]. Previous work hypothesises that Gestalt principles find roots in the biological design of auditory perception systems [9] and are therefore expected to be culture-independent.

Evidence demonstrates that both Gestalt principles and statistical learning based on long-term exposure to Western music influence pitch predictions in a musical cloze task, where participants sing the note that they expect to come next given the beginning of a melody [4]. However, Gestalt-compatible melodies are also more statistically likely in ecological music examples, making it difficult to quantify the relative contributions of Gestalt and statistical regularities to the placement of expressive accents. Here, we sought to investigate whether Gestalt and short-term statistical learning play equal roles in performed musical expression, and how they interact with listener preferences for performed accents. To do so, we designed a controlled musical system where Gestalt accents and IC accents do not coincide, and probed whether listeners prefer stimuli with the Gestalt-compatible dynamic accents, or statistically-motivated dynamic accents.

### The role of statistical learning

Shannon’s principles of efficient communication [10] have profoundly influenced the study of human communication signals such as language and music. The implementation of efficient communication principles are well-documented in language and music utterances, in particular with regard to *information content* (IC). IC, also referred to as *surprisal*, is a measure of how unexpected an element is given a previous context. IC is formally defined as:

$$\begin{array}{c} IC=-{\text{log}}_{2}\;p\left(x|c\right), \end{array}$$ (1)

where *p* is the probability, *x* refers to the outcome, and *c* refers to the preceding context. Events that are less expected in a given context have higher IC values, and are considered more informative in communication. Efficient communication principles posit that more surprising stimuli have a greater amount of resources allocated to it; this principle has been supported by empirical work showing that surprising words tend to be longer [11].

Computational models show that musical scores exhibit a wide range of IC values for musical events such as harmonic [12, 13] and pitch transitions [14]. Analyses of corpora of Western Classical music show that composers tend to assign high-IC chords to longer rhythmic values [5, 15]. In music, the Uniform Information Density theory posits that temporal emphasis on high-IC elements ensures that information is presented at a uniform rate throughout a stimulus, averting moments that are either uninteresting (IC too low) or overwhelming (IC too high) [15]. Uniform Information Density was first developed in the context of language [16]. Previous research in language demonstrates a three-way relationship between information, length, and frequency: utterances with high information are longer in duration [17], longer words tend to be less frequent [18], and less frequent words are by definition surprising. The smooth signal redundancy hypothesis [19] gives an evolutionary reason for this relationship, positing that an inverse relationship between redundancy and duration of syllables allows articulatory efforts to be more energy-efficient.

Performances by skilled musicians further increase the emphasis on surprising elements using performed accents [5]. This can take the form of slowing of tempo on chromatic chords (i.e., chords that deviate from the current key that are highly unexpected) [20], during less predictable melodic structures [21], and at departures from harmonic resolutions, when the predictability goes from high to low [22]. The increased dwell time on uncertain moments also extends to passive listeners [23].

However, much of the previous work on information-theoretic principles of accentuation have focused on temporal accents. Here, we investigate another dimension of performed accents: the *dynamic* accent consisting of a note played at a higher volume than its neighbours. Dynamic accents have been shown to be correlated with highly surprising moments in Western music [5] and may facilitate rhythm perception [7, 24]. Yet no studies have directly examined the relationship between the statistical properties of a novel grammar and the aesthetic appreciation of accentuation, independent of rhythm.

### The role of Gestalt principles

Gestalt principles refer to a small set of simple, intuitive rules that govern some aspects of melodic structures and hence predict melodic expectations and aesthetic preferences. It is thought that some Gestalt preferences have roots in biological auditory processing: the preference for Gestalt-compatible melodies with successive notes that are close in pitch (i.e. *conjunct* melodies) may be related to source separation in auditory scene analysis [25]. Conjunct melodies are also easier to parse into a cohesive stream and facilitate perceptual separation of different voices in a polyphony [26]. Furthermore, prominent melodic features such as peaks and valleys are thought to serve as cognitive landmarks, helping listeners recognise melodies more easily [27]. Arch-shaped or u-shaped melodic structures, which give rise to these peaks and valleys, are prevalent in Western melodies [28].

Melodic contour is a strong contributing Gestalt factor to immanent accents. Thomassen [8] found that contour is the best predictor of whether listeners perceive an accent on a note: peaks and valleys of a melody are often perceived as accented even when the absolute volume of the note is unchanged. Explicit accentuation is also correlated with pitch, where accents are more likely to be perceived on highest pitch of a melody (termed the *treble accent*) [8], or the highest pitch in a spoken phrase [29]. In song, the peak of melodic contour often coincides with and highlights lexical stresses [30]. A peak-shaped melodic contour is therefore highly salient in auditory processing. Just as in the case of high-IC notes, musicians tend to add performed accents that further reinforce immanent accents from the melodic contour and attract the listener’s attention, for example by dwelling longer than the written note value at melodic peaks and valleys [7, 31].

### This study

In this project, we compare listener preferences in response to the dynamic accentuation of notes with high and low IC values, or notes in different melodic contours, formed by the pitch relationship between the notes preceding and following it. Evidence that musicians add performed accents based on both statistical and Gestalt principles is rapidly accumulating. However, previous work has relied on existing Classical music material, which does not allow for addressing potential confounds such as familiarity, nor interactions with well-controlled IC values. By implementing a novel, highly-controlled musical grammar in our melody corpus, our project builds on previous work in the following ways:

i) We effectively isolate melodic contour and IC from other factors like harmony, rhythmic groupings, and metrical position, and make melodic contour and IC statistically independent. In ecological examples, the frequent co-occurrence of high-IC and Gestalt-compatible moments makes the contributing role of one musical factor difficult to isolate from the others.
ii) We investigate whether aesthetic accentuation preferences related to statistical learning can develop over the course of a short listening period.
iii) We control for long-term cultural biases by using a novel corpus of melodies based on a non-Western scale and an arbitrary but carefully controlled artificial grammar. Additionally, we evaluate the effect of musical training on the development of short-term preferences.
iv) We control for possible confounds related to performer skill (e.g. singing a high note may induce accents related to effort) by investigating aesthetic preferences from the perspective of the listener in a controlled musical setting.

Our results indicate that Gestalt principles, particularly melodic contour, are the main drivers of aesthetic preferences. For Western-trained musicians, we also found that statistical features, here implemented as the IC values of notes in the melodies, have no influence on aesthetic preferences in an unfamiliar musical system after 15 minutes of exposure.

Altogether, this work implements domain-specific principles of information processing to establish that Gestalt principles are the principal determinants of accentuation preferences in listeners. This result further highlights the relevance of a cognitive approach for various aspects of music perception, including aesthetic preference.

## Materials and methods

### Construction of melodies from an artificial grammar

We generated novel melodies using an artificial grammar based on a first-order Markov chain to manipulate IC and melodic contour while tightly controlling all other melodic properties. Seven different pitches were used in the construction of the melodies (six distinct pitches of the hexascale described below, in addition to the first scale degree repeated one octave higher).

**Fig 1:**
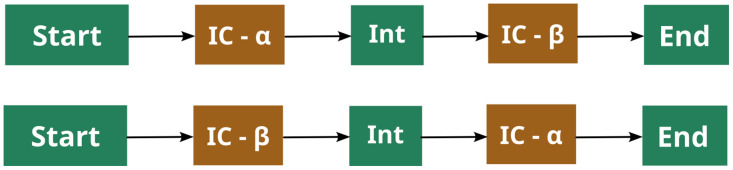
Melody modules. The two potential modular constructions of melodies used in the exposure phase.

**Fig 2:**
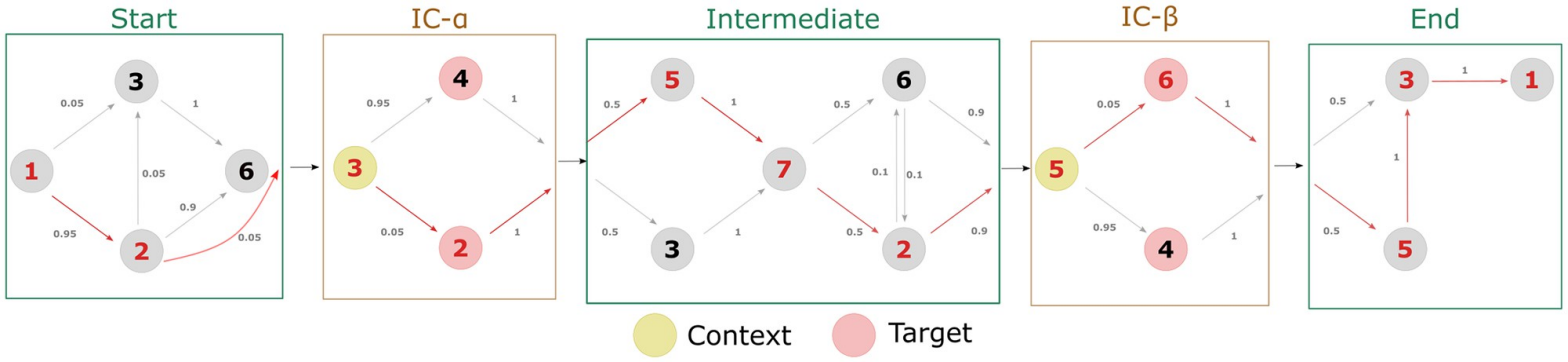
Example of melody construction. Grey arrows show all the possible paths defined by Markov chains in each cell. Red arrows indicate one possible path of a potential melody, in which there is a low-IC transition and valley-shaped contour at the first target note and a low-IC transition and peak-shaped contour at the second target note.

Sequences consisting of a string of numbers between 1 and 7 were modularly constructed as illustrated in Fig 1, with each cell following a predefined first-order Markov chain. The two IC cells (Fig 2) each contained a ‘context’ note and two possible ‘target’ notes which occur immediately after the context note. One of the target notes occurred with high probability (0.95) given the context, and the other with low probability (0.05). The start, intermediate, and end cells served to provide filler notes to create more ecological melodies, as well to vary the metrical position of the target notes and increase the variety of melodies generated.

Each IC cell had a different context note. Furthermore, in the IC-*α* cell, a target note descending relative to the context was high-IC while the ascending target note was low-IC; the inverse was true for the IC-*β* cell. This construction allowed different surrounding contours to be tested, and allowed the influence of contour and IC on accent preferences to be separated. By ensuring that the possible context-target note pairs never occurred outside the IC cells, the IC of the target note was controlled. Furthermore, the relative frequencies of each target note over the entire corpus did not greatly differ (S1 Fig), to avoid confounding IC with the frequency of the note. As noted in Fig 1, the order of the IC cells was reversed at random, which varies the metrical position of target notes (S2 Fig) while increasing the variety of the melodies.

The grammar (Fig 2) was designed so that the interval between two consecutive notes did not exceed four scale degrees, since large leaps are salient events according to Gestalt principles. Repeated notes were also potentially salient events and were avoided in the grammar. Furthermore, the interval between context and target notes was always only one scale degree. The length of the melody ranged between 9 and 14 notes, typically with 2–3 notes in each cell. The distribution of lengths of the melodies used in the exposure phase, as well as the breakdown of lengths by module, are included in the supplementary materials (S3 Fig). An example of how a melody was generated by taking one of the possible paths through the Markov grammar is shown in Fig 2.

**Fig 3:**
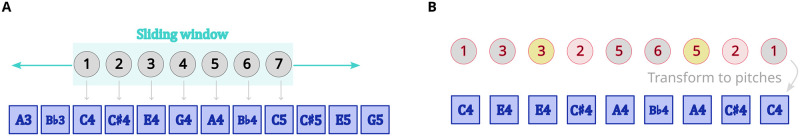
Assigning scale degrees to musical pitches. Examples of A: a scale-degree-to-pitch assignment scheme determined before the beginning of the experiment based on the position of the sliding window, and B: a sequence of scale degrees being translated into pitches according to the scheme in A.

Numerical sequences generated from the grammar were mapped to pitches using a sliding window as illustrated in Fig 3. Pitches used were determined by an asymmetrical hexascale (i.e, scale composed of 6 notes), known to elicit rapid statistical learning after only a short exposure [32]. The tonic pitch (scale degree 1) determined the position of the sliding window and thus the pitch of all subsequent scale degrees. The tonic pitch was consistent throughout the experiment for each participant, but could be changed between participant groups. The mapping varied between participants to control for differences in interval sizes between scale degrees.

Melodies were generated in Python as lists of pitches before being converted to MIDI files using the music21 library [33]. MIDI melodies were rendered as mp3 files, where each pitch was a tone on the piano, using command line tools on MacOS.

### Participants

80 participants were recruited from the Prolific.co website. Recruitment was restricted to USA or UK residents since the study was conducted in English. Participants answered three soundcheck questions consisting of identifying the melody that contains one accented note from a set of two otherwise identical melodies. Only participants who answered at least two out of the three questions correctly were invited to proceed to the experiment. Of the 78 participants (40 males, median age: 37 years; standard deviation: 11.5) who completed the experiment, 2 were excluded for continuing the experiment despite failing the soundcheck questions, 3 were excluded for not learning the artificial grammar (as described in the grammar learning test below), and 1 was excluded for systematically answering the forced choice questions before the end of the stimulus (S4 Fig). Between 10 and 13 participants were assigned to each tonic listening condition as detailed in the pitch assignment above.

Information about musical experience was collected in a voluntary survey at the end of the experiment (S5 Fig). Musicians were defined as participants who reported having followed 5 or more years of formal music lessons (at a private studio, at a Conservatory, or at a University). All other participants were defined as non-musicians.

#### Informed consent

The title page of the online experiment read as follows: “Welcome! Please read the following instructions carefully. You will be asked to listen to a series of melodies and answer some questions about what you hear. We will also collect demographic information about you and your musical background. Your participation is voluntary and you can exit the experiment at any time (however, you need to complete the experiment to receive compensation). The data collected is completely anonymous. By clicking ‘continue’ and proceeding, you provide your consent to participate.” Participant recruitment occurred from May 31 to June 3, 2024, and all recruited participants completed the experiment during that period. A compensation of 8 GBP was awarded to each participant upon completion, aligning with the recommended rates on Prolific.co. The median completion time for the experiment was 31 minutes. The experiment is covered under the authorization provided to the project “Le langage et les capacités cognitives connexes—Linguae n°20–733” by the Comité d’Évaluation Éthique de l’INSERM.

### Online experiment

The online experiment was written in JavaScript using the jsPsych library [34]. Participants were directed to the cognition.run website (an online experimental platform for jsPsych), where all pre-generated audio files used in the experiment were uploaded.

#### Exposure phase

The exposure phase consisted of passive listening trials, and two types of active trials: liking-rating, and attention trials. In each passive listening trial, participants listened to a random set of four exposure melodies generated according to the grammar outlined above. No response was solicited and the experiment proceeded automatically to the next trial at the end of the stimulus. Other types of trials were interspersed randomly throughout the exposure phase; on average, for every two passive listening trials (equating to eight melodies), there was one active trial. A total of 180 melodies were presented during the passive trials.

The occasional liking-rating and attention trials served to maintain participant engagement, but participant responses were not used in the analysis. In attention trials, participants were asked to identify the shorter melody from a set of 2 melodies. The difficulty of the attention trials ranged from ‘easy’ (7 vs 14 notes), ‘intermediate’ (7 vs 9 notes), and ‘hard’ (12 vs 14 notes) (S6 Fig). The longer melodies (12–14 notes) were generated using the same grammar as exposure melodies. The shorter melodies (7–9 notes) were generated using the forced choice grammar described below. In liking-rating trials, participants listened to one exposure melody and were asked to rate how pleasant they found the melody on a scale of 1–7 (S7 Fig).

#### Grammar learning test

During the learning test phase, participants listened to 16 melodies. After each melody, they were asked to rate how similar the melodies were to the melodies that they had heard in the exposure phase, on a scale of 1–7. Eight of the melodies presented were ‘grammatical’, drawn from the same pool as was used in the exposure phase. The other 8 were ‘agrammatical’, following the same construction as the grammatical melodies but with the pitch assignment of scale degrees 5 and 6 inverted.

#### Forced choice tests

Melodies in both forced-choice tests follow a similar construction as the exposure melodies. However, only one IC cell (chosen at random) is employed, and the intermediate cell is eliminated. As a result, there is only one target note in the melody, and the overall melody is shorter (7–9 notes), facilitating comparison between the two forced-choice alternatives. Furthermore, the target note was always found near the middle of the melody S8 Fig.

**Fig 4:**
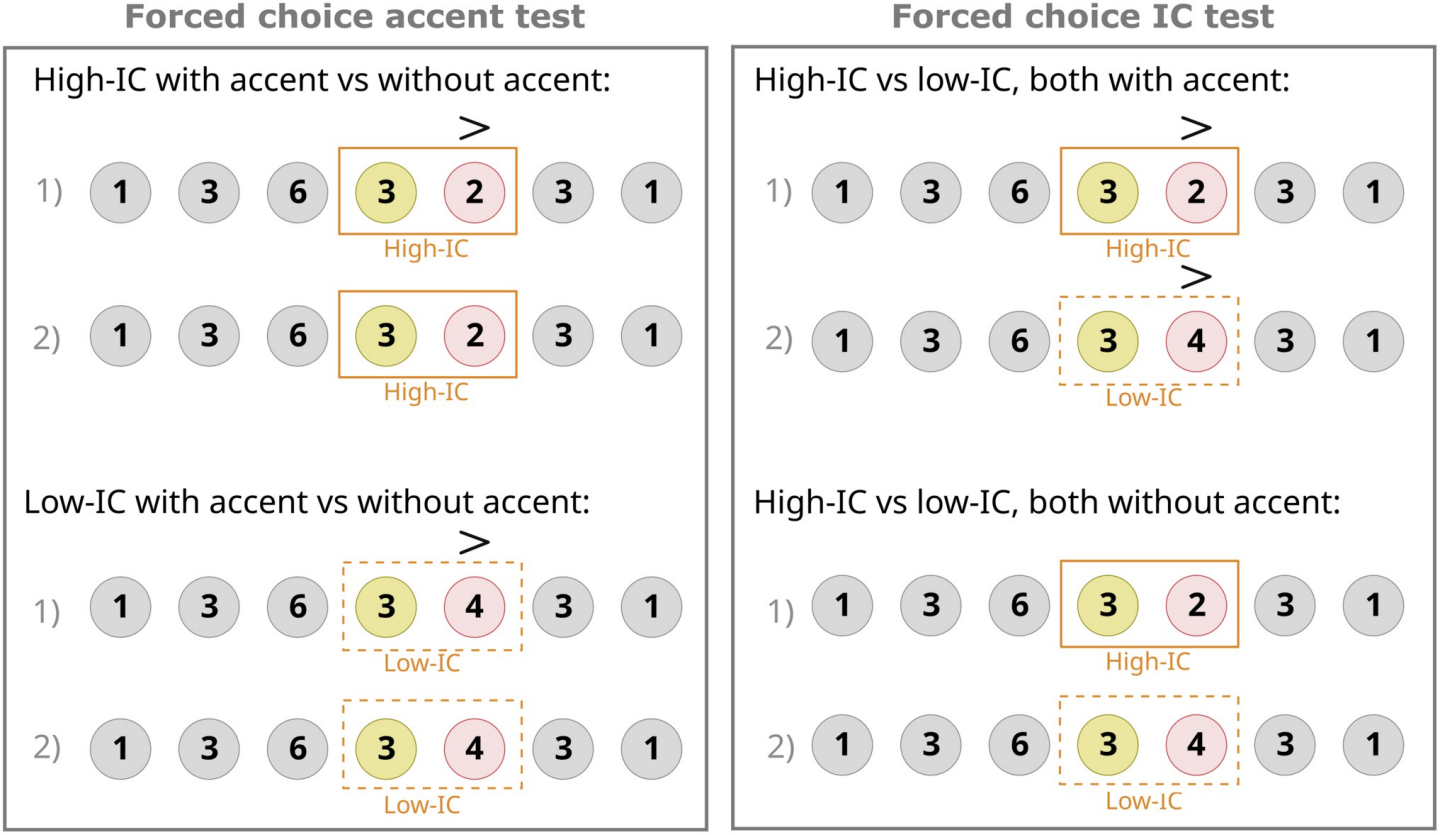
Forced choice test. Examples of the four types of forced-choice tests given to participants.

Participants completed both types of tests (described below) in randomised order; in total, each participant answered 16 questions of each type. The types of forced-choice tests are illustrated in Fig 4.

*Accent preference test*. Participants were presented with two melodies that were identical, with the target note accented in one instance and no accent in the other. They were then asked to choose which melody they preferred. In half of the questions, the target note was high-IC, while the other half had low-IC notes.

*IC preference test*. Participants again chose between two otherwise identical melodies that differed in the target note. In one instance, the target note was high-IC given the context; in the other, it was low-IC. For half of the questions, the target note was accented, while the other half had no accent.

### Data analysis

Within-subjects t-tests comparing similarity ratings for grammatical melodies and agrammatical ones were run separately for musicians and nonmusicians to assess the extent of grammar learning.

To examine the relative roles of IC and melodic contour in predicting whether listeners chose the accented versions of the melody in the accent preference forced-choice task, we fit a generalised mixed-effects model to responses in the accent preference test, with musicianship, melodic contour, and IC as variables. Melodic contours were defined as one of the following: valley (both preceding and succeeding pitches are higher than the target note), peak (both preceding and succeeding pitches are lower), ascending (preceding pitch is lower and succeeding pitch is higher), or descending (preceding pitch is higher and succeeding pitch is lower).

To examine whether accenting the target note influences the choice of high- vs, low-IC version of the melody in the IC preference forced-choice task, we fit a generalised mixed-effects model with accent as a dependent variable.

Statistical analyses were performed using the Statsmodels package for Python [35] and lme4 package for R [36].

## Results

### Transition matrix

**Fig 5:**
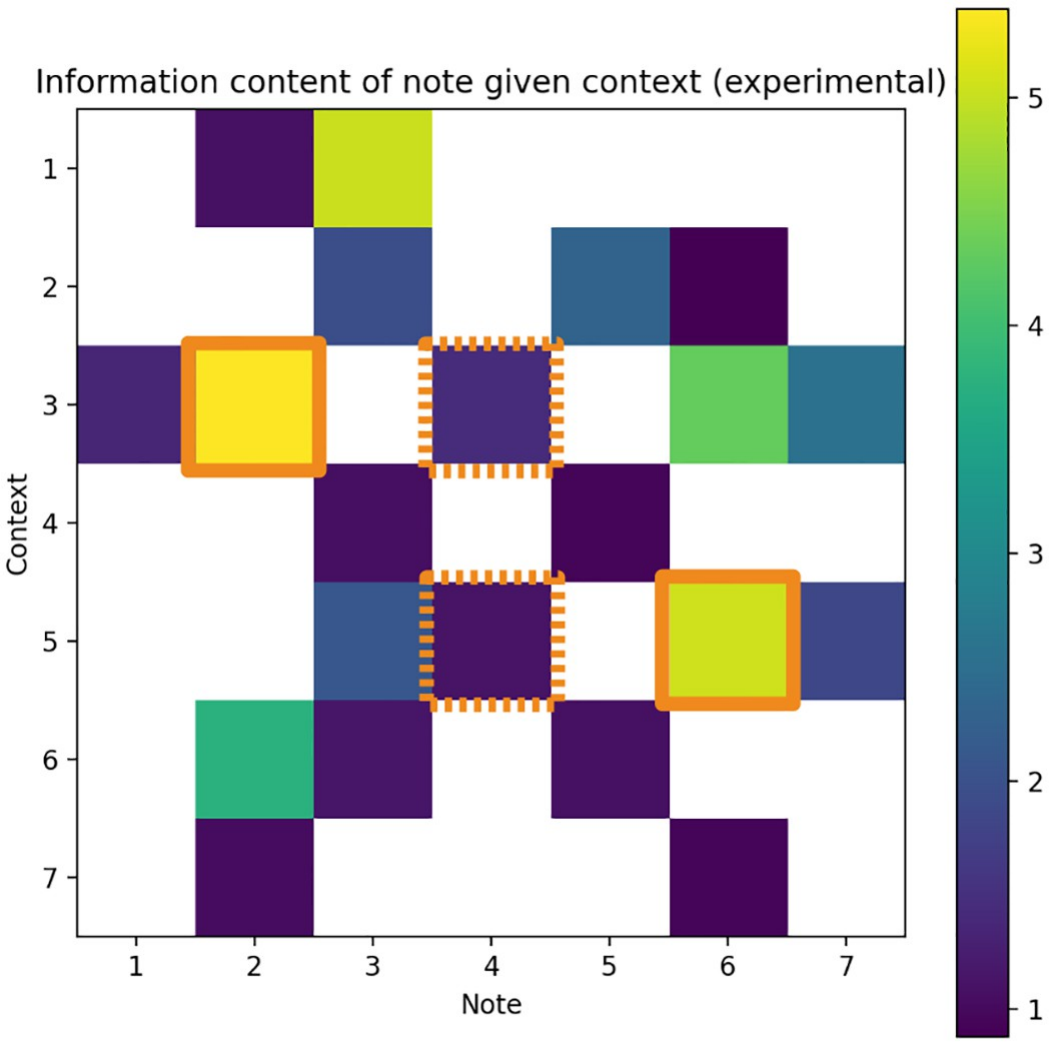
IC of context-note transitions computed over the corpus. Axes indicate scale degrees, solid boxes indicate high-IC target notes, dotted boxes indicate low-IC target notes.

The IC of each context-note transition was computed over the generated corpus to ensure that target notes matched the expected IC values. The low-IC context-target pairs (5 → 4 and 3 → 4) had IC values of approximately 1, while high-IC context-target pairs (5 → 6 and 3 → 2) had IC values of approximately 5–6 (Fig 5).

### Grammar learning

**Fig 6:**
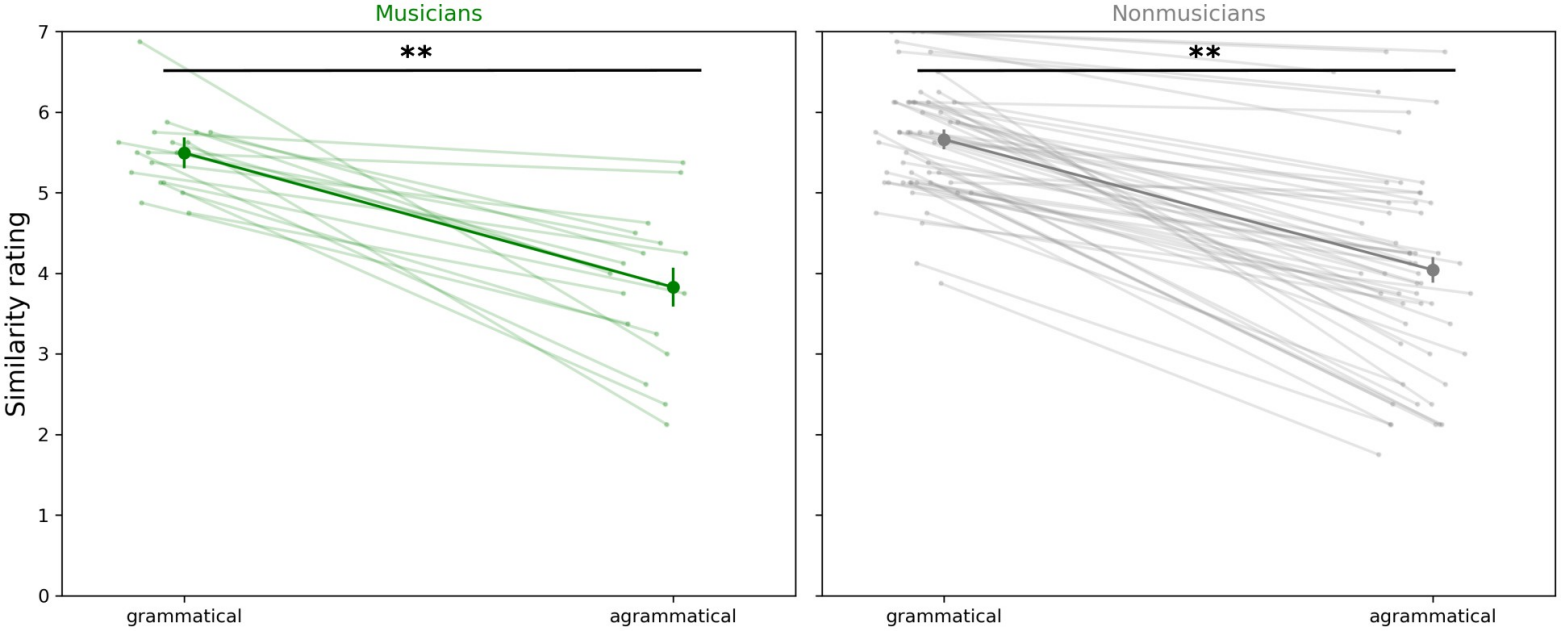
Within subject ratings of melody similarity to corpus for grammatical and agrammatical melodies for musicians and nonmusicians. Faint lines indicate individual participants, bold lines indicate mean ratings, error bars indicate 95% confidence interval.

75 of 78 participants (96%) gave lower mean similarity ratings for ungrammatical melodies compared to grammatical melodies. A within-subjects *t*-test shows an overall lower rating for ungrammatical melodies than for grammatical melodies for both musicians (*t* = 11.126, *p* < 0.001) and non-musicians (*t* = 19.530, *p* < 0.001) (Fig 6).

### Forced choice accent test

**Fig 7:**
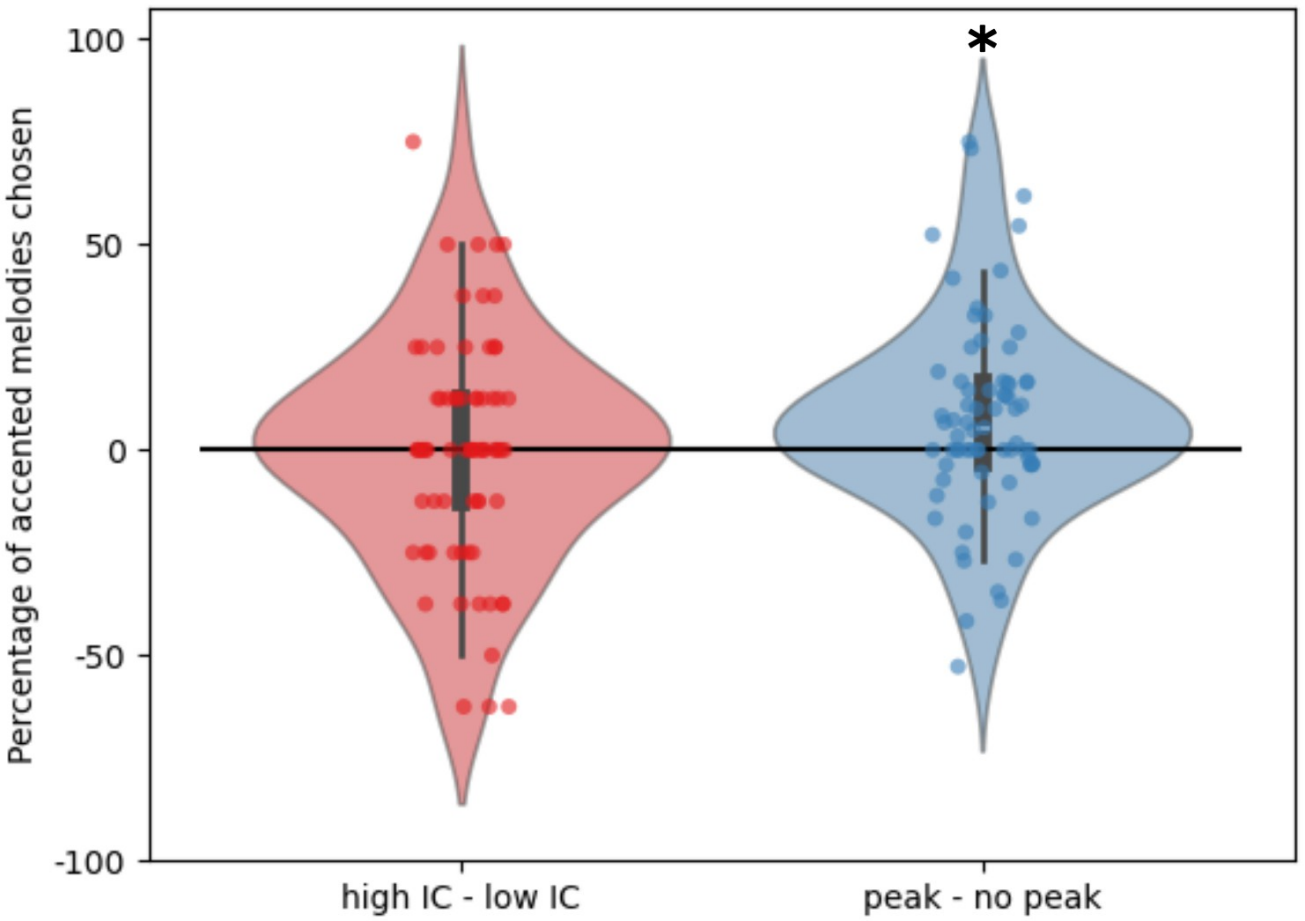
Differences in the percentage of forced-choice tests in which the accented version of the melody was chosen for conditions of high *vs* low-IC and peak *vs* no peak. Points indicate individual participants. White bars indicate the mean.

The percentage of low-IC melodies in which the accented version was chosen was compared to the percentage of high-IC melodies in which the accented version was chosen by subtracting the former from the latter. The percentage of accented versions chosen when the contour was a peak versus a non-peak was computed in the same way. Across all participants, a 1-sample *t*-test showed significantly more accented melodies were chosen when there was a peak (*t* = 2.600, *p* = 0.011) and no significant difference in the percentage of accented melodies chosen between high- and low-IC conditions (*t* = -0.0533, *p* = 0.958) (Fig 7).

**Fig 8:**
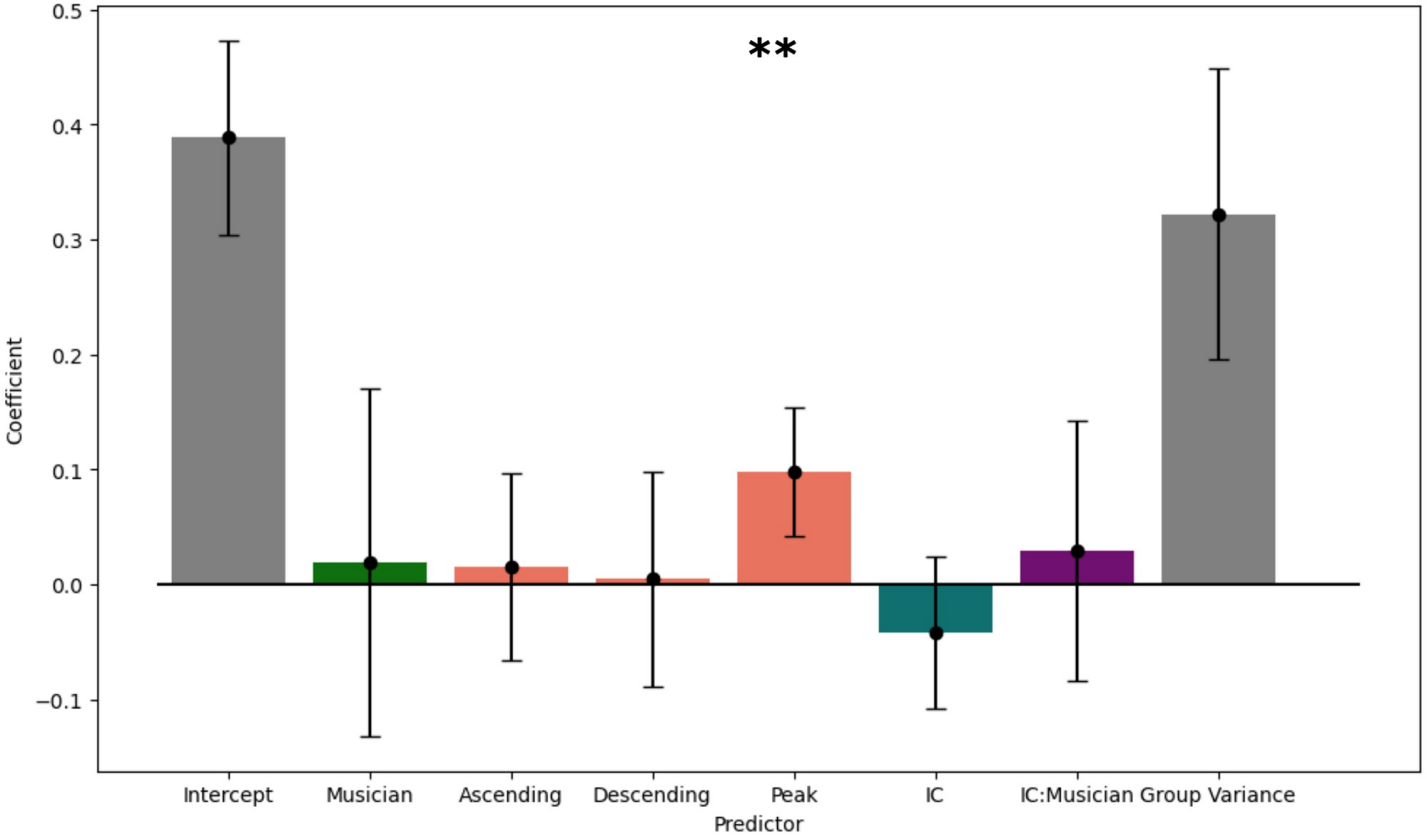
Mixed-effects model showing the effect of IC, musicianship, and melody contour on whether an accented version of a melody is chosen. Participants are included as a random effect. Points show coefficients; error bars show CI 95.

A generalised mixed-effects linear model was fit using the limited-memory BFGS (L-BFGS) algorithm with IC, contour, musicianship, and interaction between IC and musicianship as categorical fixed effects and the participant as a random effect. The model shows that a peak contour significantly predicts whether an accented version of a melody is chosen (*p* = 0.001) (Fig 8). Other coefficients are not significantly different from 0 (*p* > 0.05). Removing (i) contour from the model resulted in a significantly worse fit, while versions of the model in which we (ii) removed IC as a fixed effect and (iii) removed musicianship as a fixed effect did not significantly change model performance (Table 1). Detailed configurations and results from these models are available in the supplementary materials.

**Table 1:** Comparison statistics for full and reduced generalised mixed-effects models.

Model parameters	BIC	*χ* ^2^	p-value (vs. full model)
Full	1533.067		
(i) -contour	1524.027	12.348	0.00628
(ii) -IC	1527.268	1.330	0.249
(iii) -musicianship	1526.347	0.409	0.522

### Forced choice IC test

**Fig 9:**
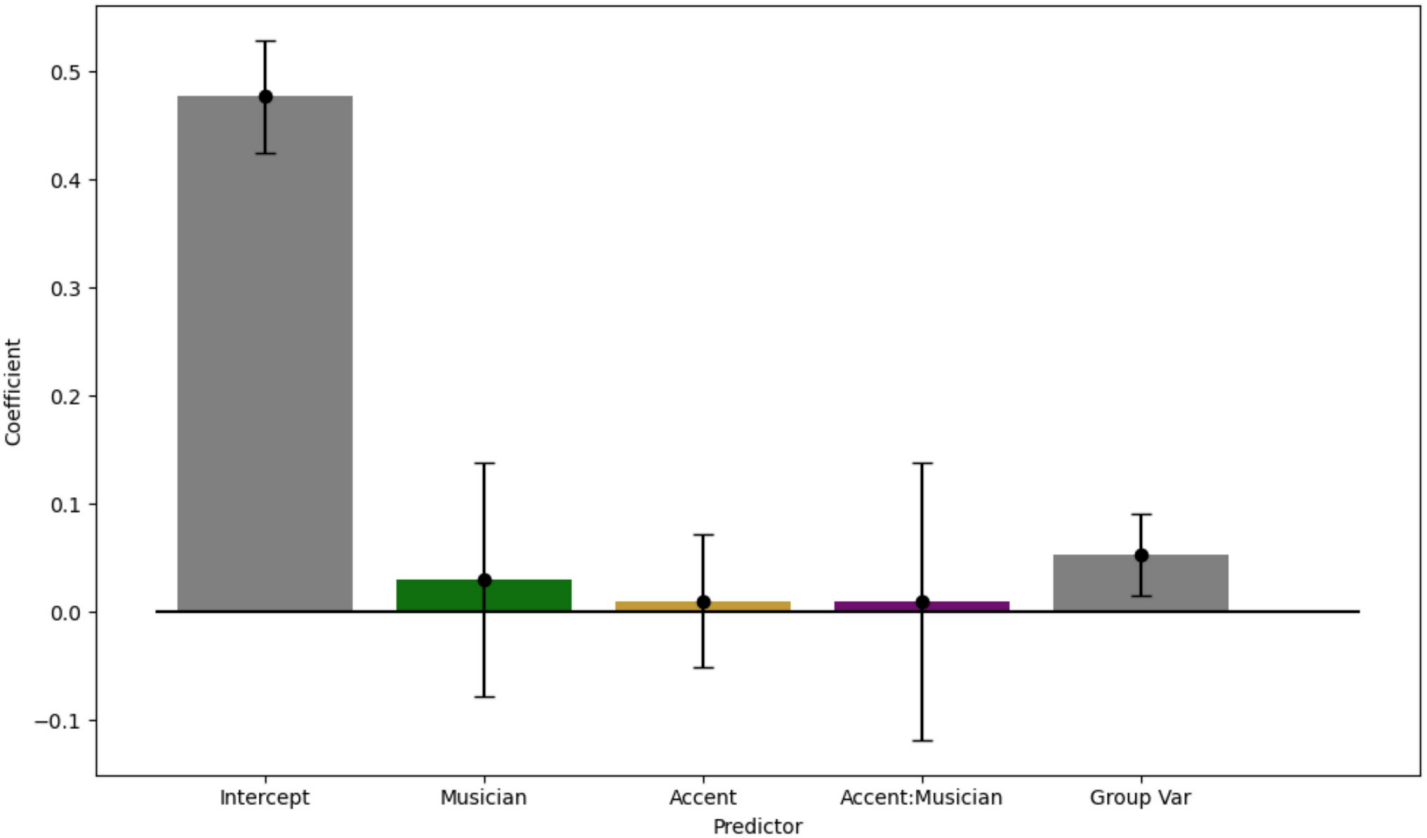
Mixed-effects model showing the effect of musicianship and accenting the target note on choosing a high-IC version of a melody. Participants are included as a random effect. Points show coefficients; error bars show CI 95.

A generalised mixed-effects model was fit using the same method as above, with musicianship, the presence of an accent on the target note, and interaction between the two as fixed effects, to assess the effect of various predictors on whether a high-IC melody was chosen over a low-IC one that was identical except on the target note. None of the predictors affected whether the high-IC melody was chosen (*p* > 0.05) (Fig 9).

## Discussion

This project aimed to investigate the role of melodic expectations in aesthetic preference during music listening. We tested the hypothesis that listener preferences for whether a note has a dynamic accent would be determined by both the note’s IC and surrounding melodic contour. Thus, we probe two different mechanisms of musical expectations –statistical regularities and Gestalt principles– on how they determine accentuation preferences. First, we exposed participants to a set of unfamiliar melodies generated from an artificial grammar in which designated target notes had either high or low surprisal given the context, and formed different melodic contours with the surrounding notes. The grammar was designed to control for many factors that may influence accent preferences: the melody was isochronous, metrical position of the target note was identical for both options of the forced choice test, and the accent always occurred in the middle of the melody. The overall frequency of all target notes was comparable (± 5% difference), so that only the IC of the note and its surrounding contour varied as a function of the context.

We then evaluated participants’ accentuation preferences on new melodies generated from the same grammar, in two forced-choice tasks where accent or IC are manipulated. All four possible melodic contours were present in both tasks. We ran generalised mixed-effects models to explain the influence of accent, IC, and melodic contour, and formal musical training on the forced-choice results.

### Rapid grammar learning

Artificial grammars governing the melodies were designed to be simple enough to learn while being distinct in style from common Western melodies. Notes in the melodies belonged to an asymmetrical hexascale (i.e. composed of 6 notes), where the pattern of intervals between each scale degree is distinct from that of the major and minor scales used in Western music. Although the scale structure is unfamiliar, Pelofi and Farbood [32] showed that its asymmetry allows novel artificial grammars governing melodies to be acquired after only a short exposure phase. To further facilitate learning, only pitches from the 12-tone equal temperament system were used to construct the hexascale.

Consistent previously reported results [32], the majority of participants (96%), regardless of musical ability, were able to differentiate between grammatical and agrammatical melodies –in which the pitch assignments for two of the scale degrees were reversed– after only a short exposure. The grammar learning results allow us to analyse the forced choice tests under the assumption that participants had retained sufficient knowledge of the artificial grammar.

### Melodic contour influences accent preferences for all listeners

During the forced-choice test phase, to facilitate the task of choosing between two melodies, the artificial grammar was modified to omit the intermediate cell and one of the IC cells to make the two forced-choice melodies shorter and easier to compare.

Melodic contour, as determined by the notes preceding and following the target note, were also expected to influence accent preferences. We found that in all participants, accented versions of the melody were more likely to be chosen when the accented target note is at a peak. This behaviour reinforces perceptual findings showing that the perceived volume is higher at the peak of a melody [8], and that perceived volume changes are enhanced by congruent changes in melodic contours (i.e., decreasing volume as the pitch descends) [37].

These results indicate that all listeners show sensitivity to melodic contour, preferring peaks to be accented. Such a preference is in line with previous work showing that higher pitches are perceived as more salient [8], and that salient notes tend to be accented [7, 31]. We show here that the preference for an accent on a melodic peak generalises even to novel melodies in unfamiliar genres. This finding is consistent with the tendency to accentuate melodic peaks found in analyses of music corpuses. However, our work brings a new dimension to corpus and performance analyses by focusing on the preferences of the listener rather than the performer or composer. We show that during passive listening in unfamiliar musical contexts, participants prefer accentuation practices that mirror Gestalt principles.

As expected, this preference based on contour is observed in both musicians and nonmusicians. Preferences regarding melodic contour are based on Gestalt principles that, by definition, should be common to all listeners regardless of previous musical exposure. In contrast to our statistical learning paradigm which requires a refined pitch perception ability to distinguish the intervals between successive notes, recognition of melodic contour demands less advanced musical abilities. Previous studies suggest that contour may have a more fundamental place in neural processing than intervals: both infants and adults without musical training are able to process contour information [38, 39], and contour is also present in speech prosody [40].

### IC does not influence accent preferences

Since the effect of target note IC on accentuation preferences was expected to be small, if any, due to the short exposure phase, we probed it in several ways.

The first type of forced choice task asked participants to select from identical melodies in which one melody had an accented target note and the other was unaccented. If IC plays a role in accentuation preferences, participants would be expected to select the accented version more often when the target note was high-IC. According to conventional performance practices, high-IC notes are frequently accented, and analysis of corpuses shows a direct correlation between the volume and the IC of a note [5]. We found a null effect of IC in predicting whether the accent was chosen, and showed that musicians and nonmusicians did not show different accent preferences. There was also no interaction between IC and musicianship.

In the second type of forced-choice task, participants selected between two melodies which differed in one note, with the differing target note being either high- or low-IC. In half of the tasks, both melodies were accented, and in the other half, both melodies were unaccented. More grammatically familiar melodies are generally rated as more pleasurable [41]– a result that would predict a preference for low-IC melodies if both melodies were unaccented. However, if there is a role for short-term IC in expressive accentuation, participants would prefer the high-IC melody if it was accented, since a dynamic accent is compatible with the high surprisal value of the note. In our results we found no effect of the accent in predicting whether the high-IC melody was chosen.

We reason that in musicians, the exposure period was likely not long enough to alter the accent preferences engrained through long-term musical training. Although we controlled for the effect of long-term statistical learning from musical exposure by minimizing any resemblance to Western musical structures, expert listeners may have nevertheless attempted to choose melodies where the accentuation was most compatible with common musical practice. Across all participants, the potential influence of Western music should be cancelled out by the different experimental conditions. The tonic of the melody (and thus the intervals between each scale degree, since the melody uses an asymmetric scale) was varied for each participant, and the scale structure differs from Western diatonic scales. While it is possible that musicians were influenced by note combinations in the melody outlining common chords, the different tonic conditions should cancel out potential influences. However, individual participants may have preferred the melodies most in line with common practice in their given experimental condition.

Knowledge of music theory that would accompany musical training may have also influenced musicians to listen to the corpus through a more analytical perspective. For example, in the post-experiment survey, musicians reported listening for specific notes and patterns to distinguish between the melodies while non-musicians made no comments or reported that they relied on gut feeling. The influence of this analytical perspective may also extend to aesthetic preferences in music training.

Sensitivity to IC modulations in musicians requires further investigation, but may be facilitated during prolonged exposure by a combination of enhanced statistical learning skills and better music analysis abilities. Musical training has been shown to enhance statistical learning of rhythms [42], as well as recall of melodies regardless of whether the melodies follow Western music rules [43]. Melodic memory in musicians is also susceptible to be supplemented with contextual details which may aid recall [44]. Together, the different types of enhanced musical memory may help facilitate the learning of IC values in novel melodies in the long run.

The potential effect of longer-term statistical learning in accentuation preferences among nonmusicians may depend on several factors. Electrophysiological evidence suggests that nonmusicians are less sensitive to intervals, particularly in unfamiliar melodies [45]. However, other work shows that nonmusicians are proficient in statistical learning [46, 47], although in some cases participants show electrophysiological responses to surprise without explicitly aware of the statistical structure [46]. In our study, nonmusicians were able to learn the grammar sufficiently well to distinguish grammatical and agrammatical melodies. Since agrammatical melodies are by definition highly surprising, we expected nonmusicians to also be able to tell the difference between the high- and low-IC melodies. For both musicians and nonmusicians, the question remains as to whether the ability to distinguish surprise in a melody may translate to an accentuation preference given enough long-term exposure.

### The relative importance of Gestalt principles and statistical learning in accentuation

In summary, musicians and nonmusicians did not show different preferences in any task. In separating the influences of Gestalt principles from short-term statistical learning, we show Gestalt principles, specifically a peak-shaped melodic contour, dominate accentuation preferences in melodies regardless of musical experience. Conversely, accentuation preferences based on statistical learning likely were not detected in this study.

We established that all listeners are able to learn a novel grammar in a short listening period, consistent with statistical learning studies in language which found that babies are able to learn statistical relationships between syllables after only 2 minutes of exposure [48], and adult participants predicting the melodies based on statistical relationships [4]. However, we find that mere familiarity with the grammar is not sufficient to change aesthetic preferences, showing that short-term statistical learning remains subordinate to Gestalt principles and existing statistical knowledge. In addition to being biologically ingrained as previously discussed, Gestalt principles are also continually reinforced throughout a listener’s lifetime via passive exposure to Western music which is largely consistent with Gestalt principles. Previous work shows that both musicians and nonmusicians are sensitive to musical structures [49], and we therefore expect melodies consistent with Gestalt principles to be highly over-represented in the priors of both groups. We cannot rule out that statistical learning could play a larger role in accent preferences with longer-term exposure to the novel corpus while curtailing exposure to Western music. However, such an experiment would require a long-term commitment from participants to obtain sufficient exposure to a novel musical grammar.

### Limitations and future steps

The grammar used in this experiment was designed to be very simple, in order to give participants the best chance of learning the IC values of the target notes over a short listening period. A side effect of this simple grammar is that many melodies closely resemble each other. Indeed, listening to many repetitions of similar melodies may become unpleasant when the exposure phase is 15–20 minutes long, and the ratings of melody pleasantness (S7 Fig) throughout the exposure phase reflect a decrease in liking throughout the experiment. This decreased liking is not consistent with previous studies on grammar learning [50], which further supports the hypothesis that the simplicity of the melodies derived from the grammar could not ensure an ecologically-valid music listening experience.

To obtain more ecologically-valid experimental conditions, a more complex or higher-order Markov chain grammar could be used to generate melodies. However, increasing complexity would likely have to be counter-balanced by a much longer exposure phase to ensure that participants learn the grammar and corresponding IC values sufficiently well before their aesthetic preferences can be evaluated.

We acknowledge that musical training entrenches a number of performance norms which may overshadow much of the new preferences developed through exposure to the artificial grammar. The influence of Western performance norms is likely to be especially prominent in high-level or professional musicians who report relying on explicit analysis in addition to intuition to develop interpretations of music. The potential influence of Western music is supported by musician participants reported resorting to analysis of the melodic contour or the pitch to determine whether the melody should be accented, rather than relying on the implicitly learned statistical information in the artificial grammar.

## Conclusion

This study implements a novel, highly-controlled paradigm to investigate long-standing musical principles regarding aesthetic preferences for accents that have thus far been primarily validated in analyses of existing musical corpora. Expressive accentuation preferences are investigated from the less commonly studied listener’s perspective, providing promising evidence that Gestalt principles generalise to novel musical styles to influence aesthetic preferences in listeners both with and without extensive Western musical training. Our evidence also shows that short-term statistical learning is insufficient to induce strong accent preferences. The results also build on previous work on the interaction between Gestalt principles and statistical learning in aesthetics, inviting further work exploring the effects of both phenomena on aesthetic preferences in different domains.

## Supporting information

S1 Fig Frequency of occurrence of scale degrees throughout the melody corpus used during the listening phase. (TIF)

S2 Fig Position within the melody, expressed as an index, at which target notes were found for each context-target pair during the exposure phase. (TIF)

S3 Fig Distribution of overall melody lengths and the lengths of the start, intermediate, and end cells used in the melody construction. (TIF)

S4 Fig Participant response times relative to the end of the stimulus (dotted black line) during the forced-choice test, used for exclusion of participants who responded exceptionally early or late (indicated by the white arrow. (TIF)

S5 Fig Distribution of reported headphone use and musical ability of participants. (TIF)

S6 Fig Scores on attention tests in which participant selected the longer of two melodies, separated by difficulty of the test (easy: 7 vs 14 notes; intermediate: 7 vs 9 notes; hard: 12 vs 14 notes.). (TIF)

S7 Fig Participant ratings of melody pleasantness throughout the exposure phase. Each colour represents one participant. Ratings were linearly interpolated across trials to be aligned across participants.
(TIF)

S8 Fig Position within the melody at which target notes were found for each context-target pair expressed as the index divided by the total number of notes. (TIF)

S1 File (PDF)
