# Supplementary figures and images for: Melodic contour supersedes short-term statistical learning in expressive accentuation
Source: PLoS One. 2024 Nov 25;19(11):e0312883. doi: 10.1371/journal.pone.0312883 (PMC11588220; doi:10.1371/journal.pone.0312883)

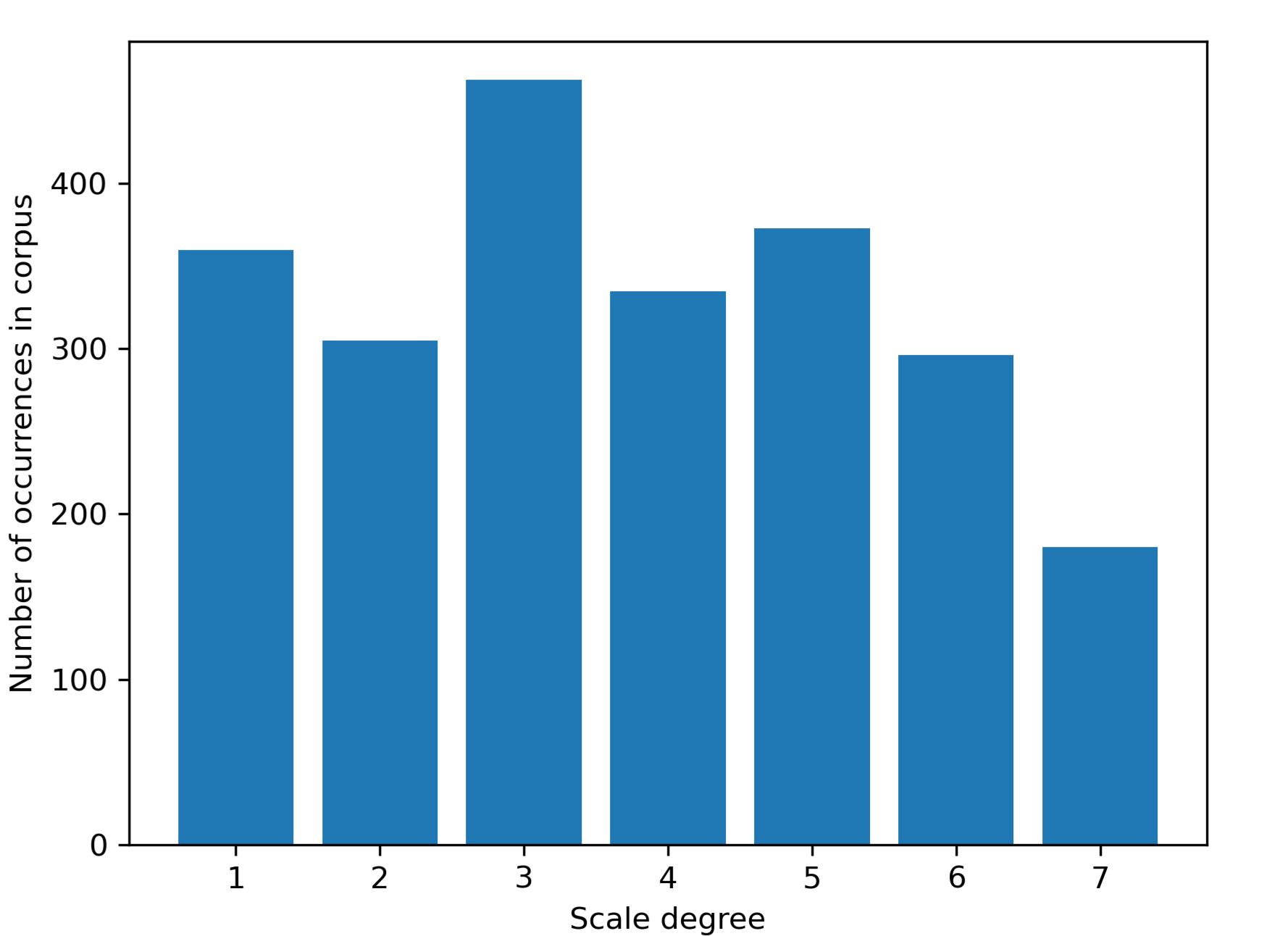

Supplement: S1 Fig — (TIF) [file pone.0312883.s001.tif]

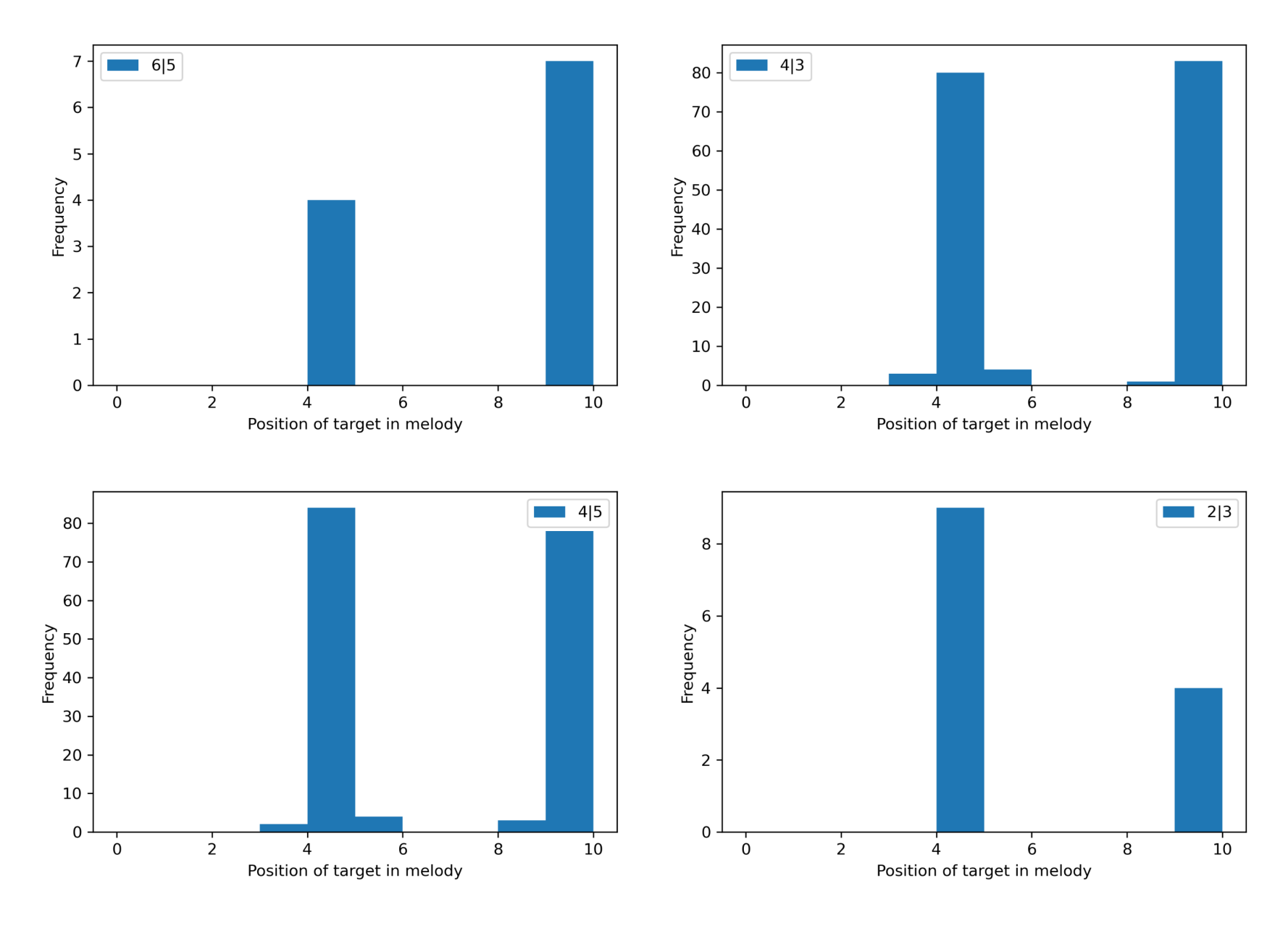

Supplement: S2 Fig — (TIF) [file pone.0312883.s002.tif]

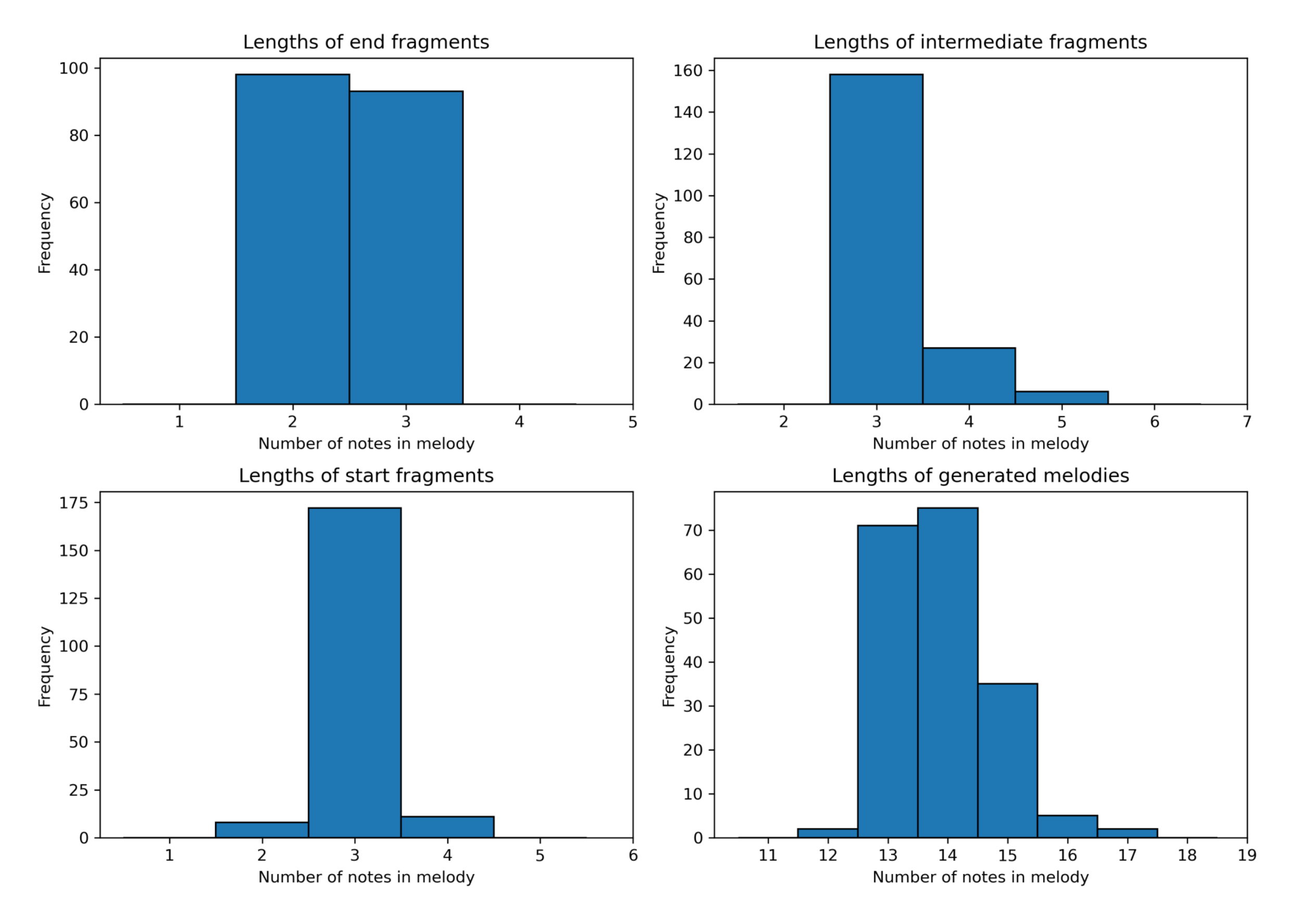

Supplement: S3 Fig — (TIF) [file pone.0312883.s003.tif]

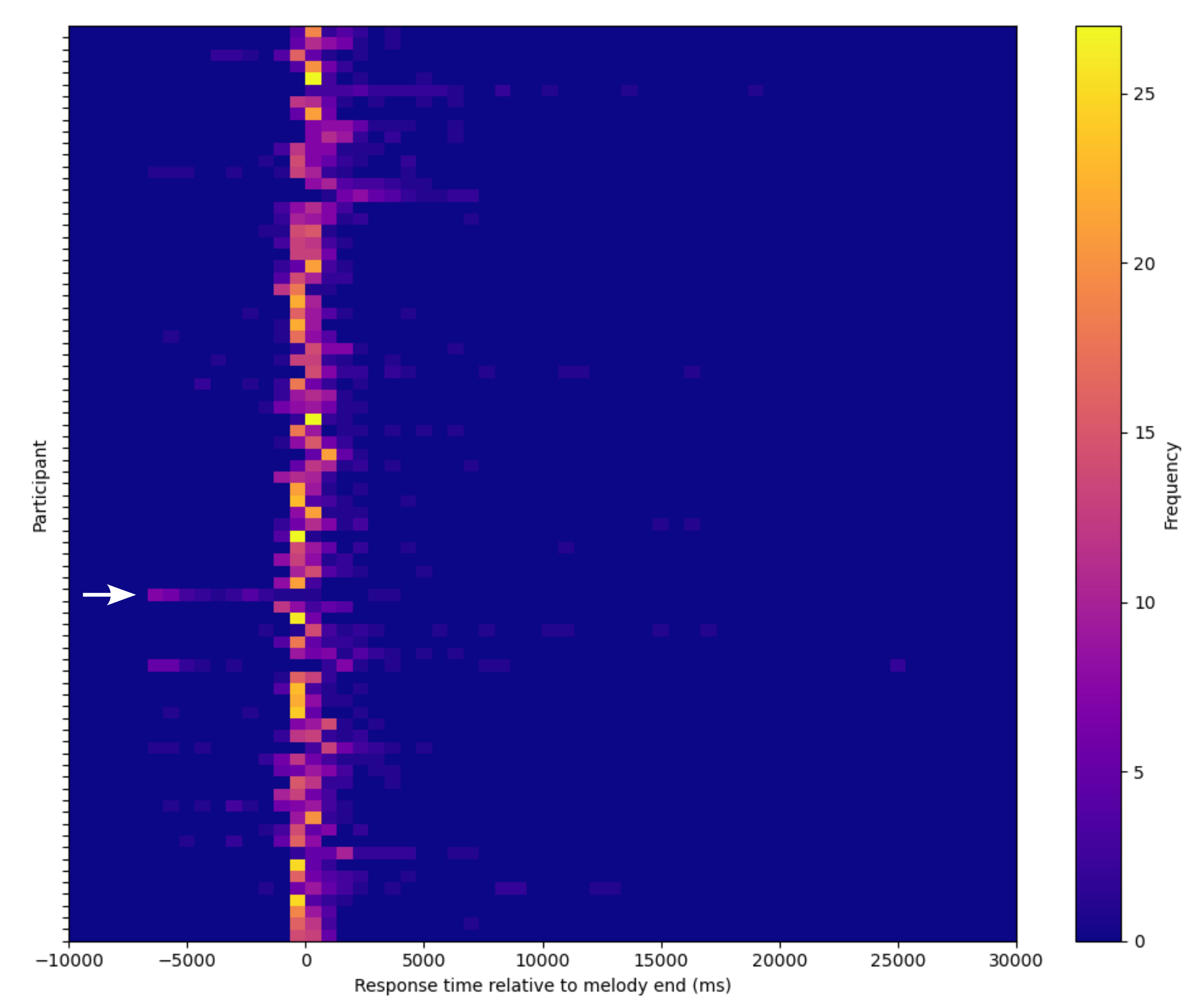

Supplement: S4 Fig — (TIF) [file pone.0312883.s004.tif]

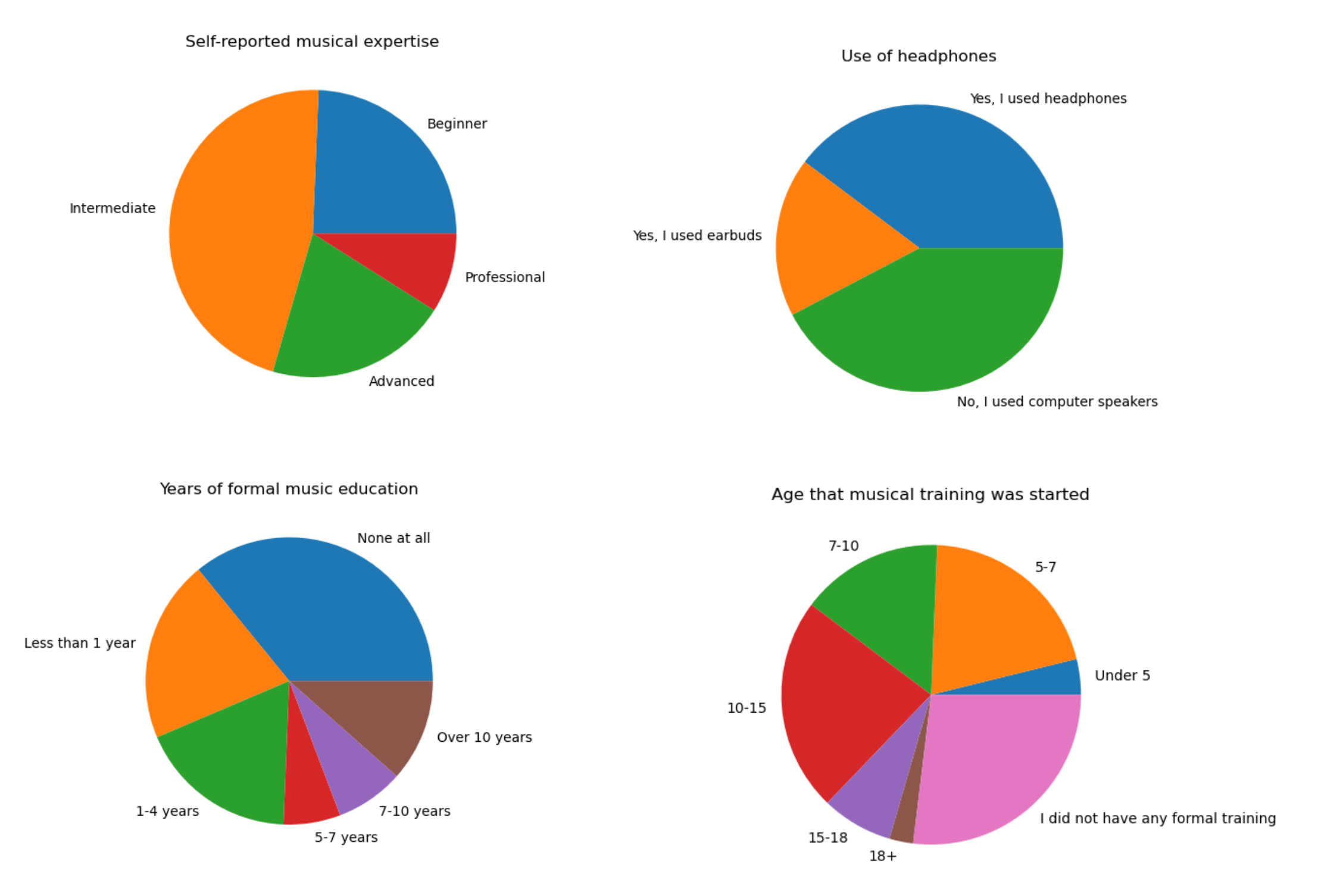

Supplement: S5 Fig — (TIF) [file pone.0312883.s005.tif]

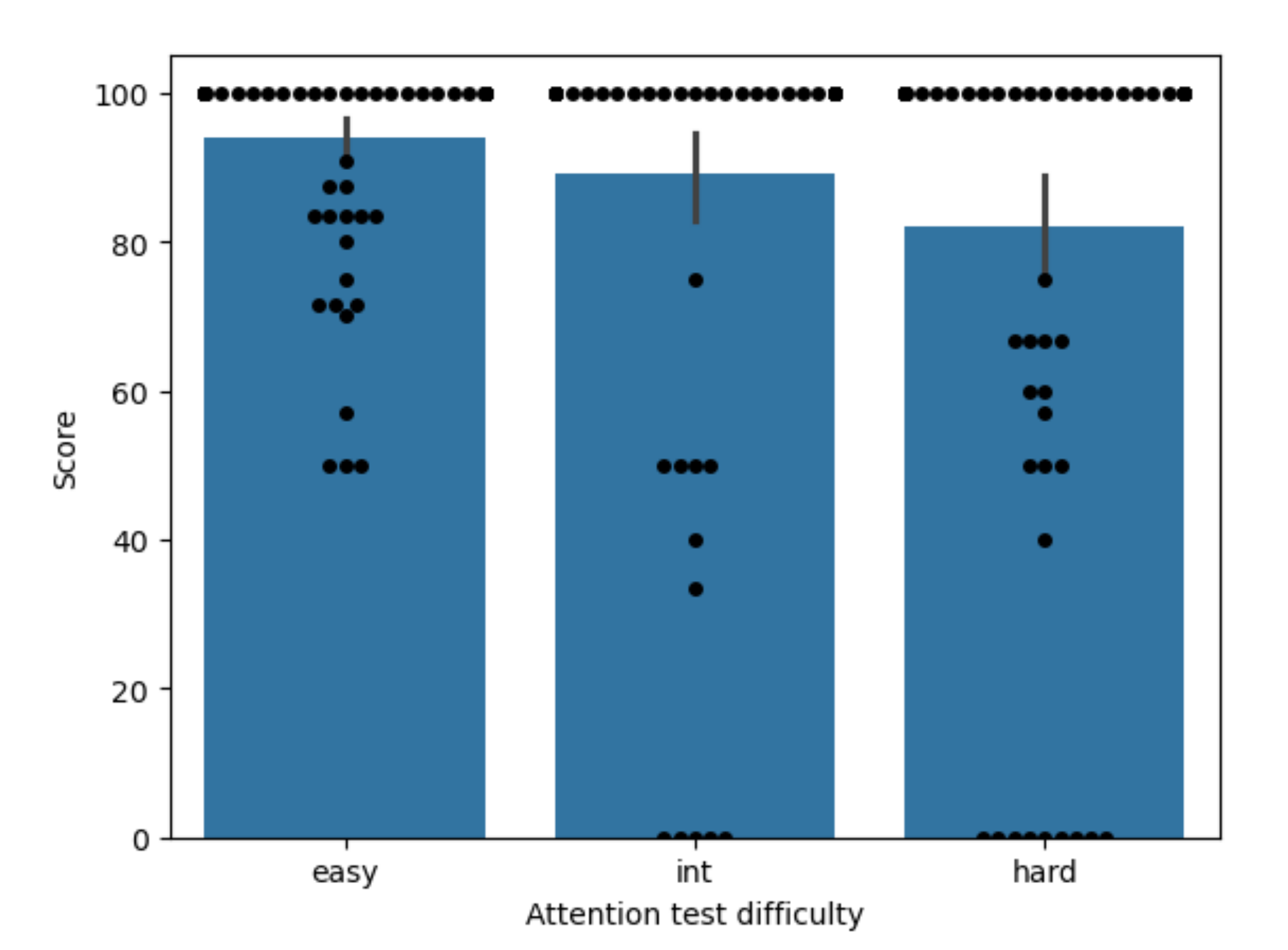

Supplement: S6 Fig — (TIF) [file pone.0312883.s006.tif]

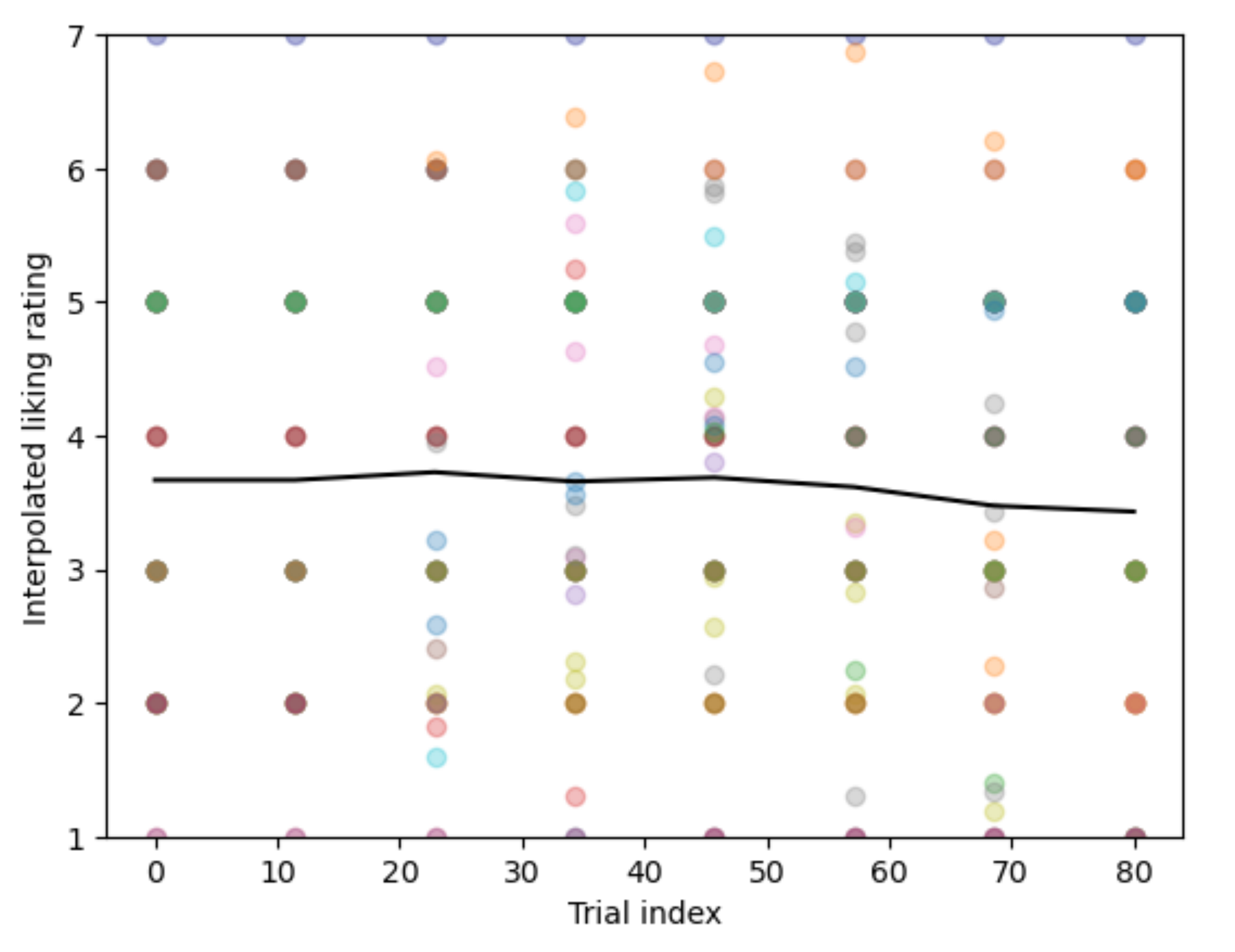

Supplement: S7 Fig — Each colour represents one participant. Ratings were linearly interpolated across trials to be aligned across participants. (TIF) [file pone.0312883.s007.tif]

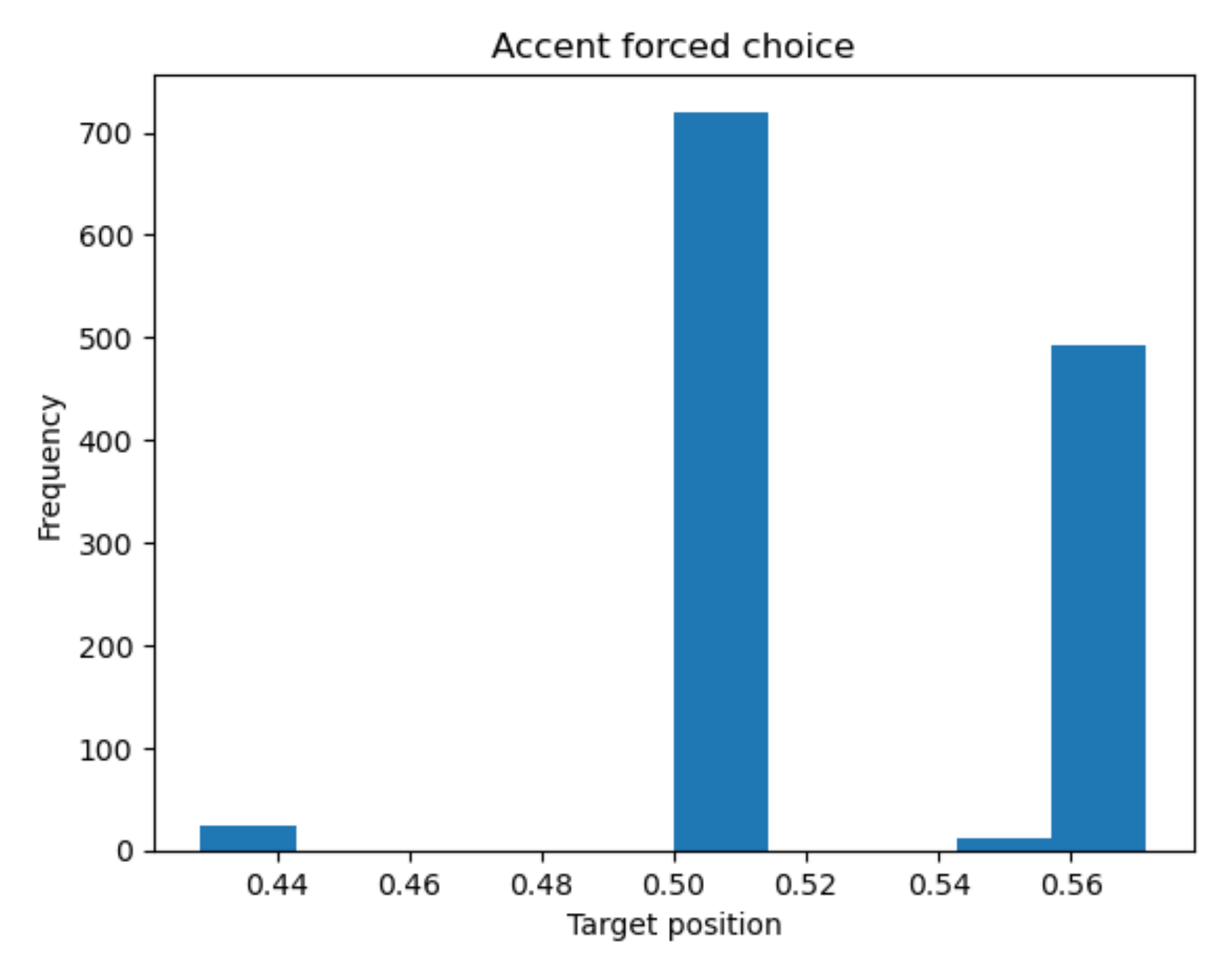

Supplement: S8 Fig — (TIF) [file pone.0312883.s008.tif]
