# Supplementary material for: Melodic contour supersedes short-term statistical learning in expressive accentuation
Source: PLoS One. 2024 Nov 25;19(11):e0312883. doi: 10.1371/journal.pone.0312883 (PMC11588220; doi:10.1371/journal.pone.0312883)
Supplement: S1 File — (PDF) [file pone.0312883.s009.pdf]

Forced choice accent

Considering only the effects of IC and contour

Mixed Linear Model Regression Results

Model:	MixedLM	Dependent Variable:	accent_chosen
No. Observations:	1248	Method:	REML
No. Groups:	78	Scale:	0.1837
Min. group size:	16	Log-Likelihood:	-794.2109
Max. group size:	16	Converged:	Yes
Mean group size:	16.0		

	Coef.	Std.Err.	z	P>|z|	[0.025	0.975]
Intercept	0.393	0.039	10.084	0.000	0.316	0.469
C(contour)[T.asc]	0.015	0.042	0.369	0.712	-0.066	0.097
C(contour)[T.des]	0.005	0.047	0.107	0.915	-0.088	0.098
C(contour)[T.inv-U]	0.098	0.029	3.416	0.001	0.042	0.154
IC	-0.035	0.031	-1.123	0.261	-0.095	0.026
Group Var	0.059	0.027				

Considering the effects of IC, contour, musicianship, and interaction between musicianship and contour

Mixed Linear Model Regression Results

Model:	MixedLM	Dependent Variable:	accent_chosen
No. Observations:	1248	Method:	REML
No. Groups:	78	Scale:	0.1839
Min. group size:	16	Log-Likelihood:	-797.6240
Max. group size:	16	Converged:	Yes
Mean group size:	16.0		

	Coef.	Std.Err.	z	P>|z|	[0.025	0.975]
Intercept	0.389	0.043	9.026	0.000	0.304	0.473
C(musician)[T.1]	0.019	0.077	0.249	0.804	-0.132	0.170
C(contour)[T.asc]	0.015	0.042	0.366	0.714	-0.066	0.097
C(contour)[T.des]	0.005	0.047	0.100	0.921	-0.088	0.098
C(contour)[T.inv-U]	0.098	0.029	3.412	0.001	0.042	0.154
IC	-0.042	0.034	-1.233	0.218	-0.108	0.024
IC:C(musician)[T.1]	0.029	0.058	0.511	0.610	-0.084	0.142
Group Var	0.059	0.028				

Considering possible effect of tonic note used

Mixed Linear Model Regression Results

```
==================================================================
===
Model:                 MixedLM    Dependent Variable:
accent_chosen
No. Observations:      1248       Method:                 REML
No. Groups:            78         Scale:                  0.1851
Min. group size:       16         Log-Likelihood:         -798.3517
Max. group size:       16         Converged:              Yes
Mean group size:       16.0
------------------------------------------------------------------
---
                           Coef.  Std.Err.   z    P>|z| [0.025
0.975]
------------------------------------------------------------------
---
Intercept                   0.417    0.032 13.008 0.000  0.354
0.479
IC:C(condition)[tonic A4]  -0.122    0.058 -2.097 0.036 -0.236
-0.008
IC:C(condition)[tonic Bb4]  0.010    0.058  0.179 0.858 -0.104
0.124
IC:C(condition)[tonic C5]   0.041    0.058  0.695 0.487 -0.074
0.155
IC:C(condition)[tonic Cs5] -0.045    0.048 -0.947 0.344 -0.139
0.049
IC:C(condition)[tonic E5]   0.076    0.058  1.300 0.194 -0.038
0.190
IC:C(condition)[tonic G5]   0.001    0.059  0.015 0.988 -0.114
0.116
Group Var                   0.057    0.026
==================================================================
===
```

Considering interaction between musician and contour

```
Mixed Linear Model Regression Results
==================================================================
============
Model:                     MixedLM       Dependent Variable:
accent_chosen
No. Observations:          1248          Method:
REML
No. Groups:                78            Scale:
0.1839
Min. group size:           16            Log-Likelihood:
-799.6541
Max. group size:           16            Converged:
Yes
Mean group size:           16.0
------------------------------------------------------------------
------------
                                     Coef.  Std.Err.   z    P>|z|
[0.025 0.975]
```

```
------------------------------------------------------------------
-------------
Intercept                              0.392    0.043  9.029 0.000
0.307  0.477
C(musician)[T.1]                       0.008    0.080  0.095 0.924
-0.149  0.164
C(contour)[T.asc]                      0.005    0.047  0.114 0.909
-0.086  0.096
C(contour)[T.des]                      0.014    0.052  0.268 0.789
-0.089  0.117
C(contour)[T.inv-U]                    0.081    0.033  2.487 0.013
0.017  0.145
C(contour)[T.asc]:C(musician)[T.1]     0.041    0.086  0.475 0.635
-0.127  0.208
C(contour)[T.des]:C(musician)[T.1]    -0.049    0.105 -0.470 0.638
-0.255  0.156
C(contour)[T.inv-U]:C(musician)[T.1]  0.072    0.067  1.075 0.282
-0.060  0.204
IC                                    -0.035    0.031 -1.141 0.254
-0.096  0.025
Group Var                              0.059    0.028
==================================================================
=============
```

Forced choice IC

Considering only the effect of accent

```
Mixed Linear Model Regression Results
============================================================
Model:             MixedLM Dependent Variable: high_IC_chosen
No. Observations: 1248    Method:             REML
No. Groups:        78      Scale:              0.2380
Min. group size:  16      Log-Likelihood:     -903.4563
Max. group size:  16      Converged:          Yes
Mean group size:  16.0
-------------------------------------------------------------
               Coef.  Std.Err.    z     P>|z|  [0.025  0.975]
-------------------------------------------------------------
Intercept      0.484     0.023  20.814  0.000   0.438   0.530
accent         0.013     0.028   0.464  0.642  -0.041   0.067
Group Var      0.012     0.009
============================================================
```

Considering possible effect of musicianship

```
Mixed Linear Model Regression Results
=================================================================
Model:                MixedLM  Dependent Variable:  high_IC_chosen
No. Observations:     1248     Method:              REML
No. Groups:           78       Scale:               0.2382
Min. group size:      16       Log-Likelihood:      -907.1326
Max. group size:      16       Converged:           Yes
```

```
Mean group size:     16.0
-----------------------------------------------------------------
                         Coef. Std.Err.   z    P>|z| [0.025 0.975]
-----------------------------------------------------------------
Intercept                0.477    0.027 17.964 0.000  0.425  0.529
C(musician)[T.1]         0.030    0.055  0.540 0.589 -0.078  0.138
accent                   0.010    0.032  0.331 0.741 -0.051  0.072
accent:C(musician)[T.1] 0.010    0.066  0.159 0.874 -0.118  0.139
Group Var                0.013    0.009
=================================================================
```

Grammar learning

Considering effect of individual melodies

```
Mixed Linear Model Regression Results
==================================================================
=====
Model:                 MixedLM      Dependent Variable:
response
No. Observations:      1248         Method:                  REML
No. Groups:            16           Scale:
2.2407
Min. group size:       78           Log-Likelihood:
-2281.4596
Max. group size:       78           Converged:               Yes
Mean group size:       78.0
------------------------------------------------------------------
-----
                              Coef. Std.Err.   z    P>|z| [0.025
0.975]
------------------------------------------------------------------
-----
Intercept                     4.066    0.082 49.568 0.000  3.905
4.226
grammaticality[T.grammatical] 1.540    0.116 13.277 0.000  1.313
1.767
Group Var                     0.025    0.014
==================================================================
=====
```
